# Delivering cellular and gene therapies to patients: solutions for realizing the potential of the next generation of medicine

**DOI:** 10.1038/s41434-019-0074-7

**Published:** 2019-04-25

**Authors:** Kris Elverum, Maria Whitman

**Affiliations:** 1 Turnstone Biologics, New York, NY USA; 2 ZS Associates, Philadelphia, PA USA

**Keywords:** Gene therapy, Gene delivery, Cell delivery, Cell therapies

## Abstract

The evolution of medicines from small molecules to proteins drove increased therapeutic benefits, and the next generation of cell and gene therapies holds tremendous promise for patients. The Food and Drug Administration approved the U.S.’s first gene therapy, Novartis’ tisagenlecleucel, and technologies like CRISPR-Cas9 are poised to create a wave of new medicines. Unfortunately, the vast majority of patients may not benefit from cell and gene therapies. At least 95% of people receive medicines only through commercial delivery, but stakeholders have struggled to develop and sustain successful business models for cell and gene therapies. This paper reviews the existing system to deliver cell and gene therapies and outlines the requirements to make them accessible to patients. Informed by interviews with experts, opportunities for improvement are identified along the patient and cell journeys, and a call to action is made for stakeholders to detail and implement change.

## The Promise and the Challenge

The evolution of medicines from small molecules to proteins drove increased therapeutic benefits, and the next generation of cell and gene therapies holds tremendous promise for patients. The FDA had 500 active investigational new drug applications involving gene therapy products in 2018 [1], and $2.3 billion in funding went to private gene therapy companies over the last decade [2]. Clinical trial results have been promising, including high rates of complete responses in cancer [3] and reversing blindness caused by specific gene mutations [4]. The FDA has innovated approval pathways, such as the regenerative medicine advanced therapy (RMAT) designation, and in 2017 the first gene therapy, Novartis’ tisagenlecleucel, received marketing approval in the U.S.

Unfortunately, approval is not enough to help patients. The vast majority of patients may not receive cell and gene therapies as stakeholders struggle with successful business models for approved medicines. At least 95% of people receive medicines only through the commercial delivery system: for example, less than 5% of cancer patients participate in clinical trials [5]. However, sales of tisagenlecleucel have underperformed expectations [6], the manufacturer of another personalized cell therapy (sipuleucel-t) declared bankruptcy [7–9], and hospitals and payers have struggled to provide access to these novel therapies [10–12].

The delivery system designed for pills and biologics urgently needs to be changed to accommodate cell and gene therapies. Changes to the privately funded, insurance-based system will not happen fast enough, and piecemeal improvements will not move the needle enough for the 39 gene therapies projected to be approved by 2022 [13].

In this paper, we seek to review the system to deliver cell and gene therapies and outline the requirements to make them accessible to patients. Informed by interviews with leading experts, we identify opportunities along the patient and cell journeys, and make a call to action for stakeholders to detail and implement change.

## Developing New Journeys

**Fig. 1:**
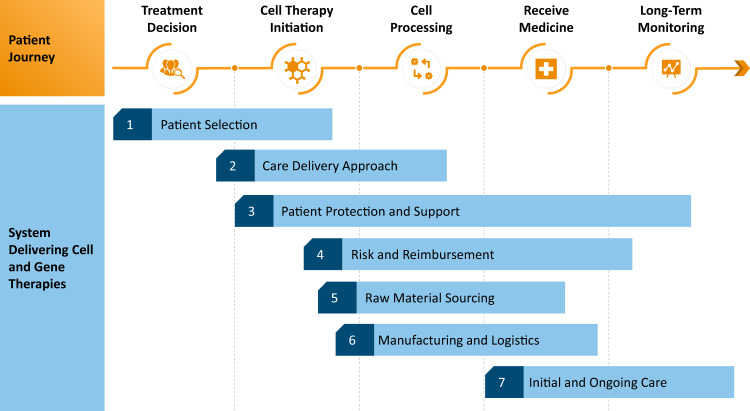
System for delivering individualized, autologous, ex vivo, cellular therapies. Patients proceed from the treatment decision to cell therapy initiation and cell processing before receiving a medicine, and then ultimately being monitored long-term. The current system to deliver medicines to patients includes seven steps, each of which is assessed in detail for challenges and opportunities: (1) patient selection, (2) care delivery approach, (3) patient protection and support, (4) risk and reimbursement, (5) raw material sourcing, (6) manufacturing and logistics, and (7) initial and ongoing care

The patient, product, and system journeys for cell and gene therapies are fundamentally different than traditional medicines. There are multiple types of cell and gene therapies: some medicines are individualized to a single patient while others apply to a group; some leverage human material and others do not; some will be engineered ex vivo and others in vivo. To best understand the system-wide challenges for cell and gene therapies, we will discuss the patient journey and supporting system for the most complex of these new medicines: personalized, autologous, ex vivo cell and gene therapies (Fig. 1), which includes medicines like the recently approved CAR-T cell therapies. Many of these challenges will apply to other types of cell and gene therapies, and therefore we believe this is an effective framework for developing the delivery system for the next generation of medicine.

### Step 1: Patient Selection

Cell therapies offer a new treatment option, but patient selection may differ for clinical trials and real-life applications, and many institutions may not have the needed capabilities. For individualized therapies, patients will need to meet the clinical criteria (indication, prior therapeutic options, performance status) as well as specific health criteria to endure the time to manufacture the product [14]. Appropriate and rapid patient selection is critical; however, standards, physician practice capabilities, and economic incentives must be addressed for eligible patients to receive treatment.

Patients will need to be treated at centers meeting the standards of organizations such as the Foundation for the Accreditation of Cellular Therapy (FACT), but the government does not directly oversee these guidelines. Individual manufacturers may also exercise control as determined by an FDA-mandated Risk Evaluation and Mitigation Strategy (REMS), which often are vague and have a wide variety of training and implementation needs. Hospitals’ management of different standards will become increasingly challenging as more medicines obtain approval. Providing regulatory oversight to FACT, and streamlining and standardizing accreditation and REMS programs by application, would decrease the effort and investment required to implement individual therapies, enabling use on a larger scale.

Many practices do not yet have the capabilities (e.g., cell handling) to provide complex cell therapies, and institutions will need to invest to deliver these technologies. However, there is a shortage in physician capacity to treat patients [15], and the fee-for-service system does not compensate for developing cell therapy capabilities. One major academic center had 60 people attend a kick-off meeting with a CAR-T manufacturer, an unsustainable approach when multiplied by many medications and manufacturers.

There are just 218 FACT-accredited institutions in the U.S., and some states lack any accredited institutions (e.g., Alaska), while others do not have institutions accredited for all transplant types [16]. Many patients will require referrals to be treated; however, local physicians may have a financial disincentive to refer patients as they may make money on pharmaceuticals through average sales price (ASP) reimbursements [17]. The Centers for Medicare and Medicaid Services (CMS) have promoted approaches that tie reimbursements to patient outcomes, but progress has been slow and new reimbursement models would ensure appropriate coordination and referrals.

### Step 2: Care Delivery Approach

The clinical delivery of cell and gene therapies is more complex and multidisciplinary than pills or biologics. Certified institutions will need a different model of patient care coordination, and manufacturers, enabled by government regulation, will have to go beyond traditional approaches to ensure patients get treatments and physicians are appropriately supported.

Cell therapy patients are likely to require complex care before, during, and after therapy. For example, prior to receiving gene-edited immune cells that remove a specific gene mutation, a patient may have their immune system ablated, putting him or her at critical risk for infections. Healthcare professionals trained in silos will need to work together, and leading institutions are establishing new cell therapy teams and approaches [18]. Institutions will need to create models that work within their structures, define new roles (e.g., expanded bone marrow transplant coordinator), and develop protocols for inpatient and outpatient care and monitoring.

Cell therapies will also require tight coordination between physicians and manufacturers, as products are manufactured for individual patients, and changes in timing impact physicians’ treatment decisions. Under pill and biologic regulations, manufacturers have separate commercial and medical functions, and do not coordinate for individual patients. Pharmaceutical companies will need to rethink the roles interacting with healthcare professionals, and regulators should reevaluate the rules, potentially using the approach for interventional medical devices as a guide.

**Fig. 2:**
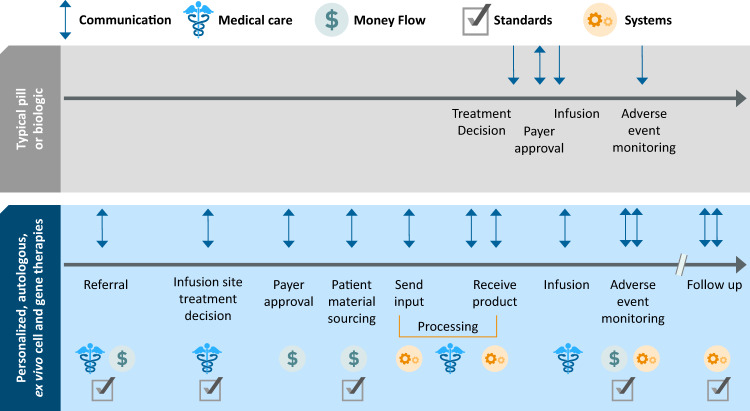
Overview of communication touchpoints and changes needed. The commercial delivery of pills and biologics primarily involves one-way decision making among few stakeholders, and much of the regulation and standards in development and manufacturing occur outside of the patient journey. Personalized cell therapies, in particular autologous, ex vivo gene therapies, have an elongated set of multi-stakeholder communication touchpoints, and the medical decisions and personal, financial decisions and money-flow, standards, and systems support are varied across the touchpoints

Delivering personalized cell therapies is complex and requires significantly more coordination between stakeholders than pills or infusions, and there are challenges in communication, medical care, money flow, standards, and systems as highlighted in Fig. 2.

### Step 3: Patient Protection and Support

The types of patients who may benefit from cell and gene therapies are diverse: a cancer patient with advanced disease, a child with a rare genetic condition whose parents must coordinate care across multiple states, or someone born with blindness that may see for the first time. Patients will go through a difficult and extremely expensive process, likely more so than for traditional therapeutics. To ensure access, payers, hospitals, manufacturers, and the government need to provide financial support and evolve the approaches to patient information.

Patients and families will likely incur significant out-of-pocket costs to go to a certified center, stay nearby, and manage a complicated life for a period of months or years. While nonprofit organizations such as Ronald McDonald House Charities provide assistance, ensuring payers provide reasonable reimbursement for all associated costs is critical. Patient support programs from manufacturers also help, and adjusting rules so services can be provided when physicians make treatment decisions (so long as they are not an inducement to prescribe) would make this process more efficient and effective.

Patient data will travel across multiple institutions and disciplines, raising the importance of a seamless transfer of information and protection of confidentiality. A center will order the therapy with protected patient information directly from the pharmaceutical company or third-party distributor. Pharmaceutical companies may need to become HIPAA-covered entities or business associates, and tailored technical solutions will need to be developed and rules adopted to cover access to information, even with broad patient consent.

### Step 4: Risk and Reimbursement

Cell and gene therapies highlight the disconnect between prices and outcomes in our funding system. Many of these therapies will be given just once, but their value may accrue over a lifetime. This disconnect makes risk-sharing reimbursement strategies critical, with government action needed to enable them.

Cell therapies will be approved as biologics and reimbursed as drugs [19, 20], which are priced per-use. Traditionally, this reimbursement approach meant payments correlated with outcomes as manufacturers continued to receive revenue only if patients stayed on therapy. The list price of tisagenlecleucel for one-time delivery was $425,000 at launch, and a recently approved adeno-associated virus vector-based gene therapy (voretigene neparvovec-rzyl) was $850,000 [21]. In some specialty therapies, such as certain cancers where novel combinations have the potential for multiyear durability, the costs may be similar to cell and gene therapies in the long run.^1^ However, the difference between paying over time versus paying the full cost in a one-time, up-front, cash outlay, regardless of outcomes, highlights the need for new reimbursement models.

The payment system also has the potential to limit hospitals’ willingness to provide cell and gene therapies. Hospital reimbursement is based on ASP plus a small percent for outpatient drugs, but for inpatient stays all costs are bundled under diagnosis-related groups (DRGs), which do not include the costs of new cell therapies. To avoid losing significant amounts of money per patient, hospitals are currently negotiating with payers for each patient, a difficult process for hundreds of childhood leukemia patients but impossible for thousands of patients with rare diseases. Separating cell therapies, or orphan drugs, from the DRG system is a debated topic and would be a simple way of removing a disincentive to treat patients with new therapies, but might create other pressures in the system.

Outcomes-based models have emerged as a potential solution in some situations. Novartis and CMS recently ended a pilot program for outcomes-based pricing for tisagenlecleucel [22, 23], however others are pursuing outcomes-based approaches, such as Spark Therapeutics for voretigene neparvovec [24]. Practical implementation challenges such as IT systems, endpoint definition and application of measurement currently pose significant barriers. Patient movement between insurance plans—20–30% of people change insurance carrier or plan each year [25]—adds further complexity and risk to the insurer business model and may be preventing good ideas. There may be short-term options for tangible steps in the right direction, such as selectively waiving “best price” and enabling different product codes based on the patients’ diagnosis or indication.

Cell and gene therapies highlight the deficiencies in the drug reimbursement system and identified short-term changes would help; however, long-term solutions require significant analysis and have been debated elsewhere [26]. Ultimately, CMS is uniquely positioned to drive meaningful change and must take a pivotal lead, as other stakeholders follow the system established by CMS for drug reimbursement.

### Step 5: Raw Material Sourcing

Autologous therapies use a patient’s material to create a personalized product, a process that involves local care providers (e.g., tissue extraction) or blood centers (e.g., Red Cross), specialized handling (e.g., cryopreservation) and niche shippers, all of which may not be affiliated with the patient’s care team. The FDA deems the starting patient material a manufacturing input, requiring companies to demonstrate quality control. Improving standards will support the growth across applications, and controls over payments should be implemented to prevent abuse.

FACT and AABB provide standards for select procedures (e.g., leukapheresis); however, efforts to consolidate guidelines have not been successful and companies’ processes often differ. Standards do not exist for an array of new therapeutic areas (e.g., regenerative medicine) and material types (e.g., tumor lysate). The result is that local centers and cell labs will struggle to manage a large number of different protocols, each of which may only be used by a few patients. Consolidating standards is a key first step, and the early establishment of standards by class of product would also improve quality oversight.

Manufacturers will pay blood centers and cell labs, just like other manufacturing suppliers. This has the potential for conflict as many of these institutions will be the same as where treatment decisions are made. Establishing transparent fair market values, and payments through third parties, will ensure that payments are not used as a means of competition and artificial profit centers are not created.

### Step 6: Manufacturing and Logistics

Patient materials move from local labs to manufacturing facilities and then to hospitals for treatment, a process requiring technology systems and quality tracking to ensure patients receive their engineered cells, as errors could have dire consequences. Patients’ cells will be engineered at certified good manufacturing practice (cGMP) facilities in closed systems that operate through production line or device-based approaches within separate suites [27, 28]. Current processes were established in a lab for small-scale experiments, and significant improvements to technology, cost, and time will be necessary.

Manufacturers will develop and manage a singular, closed-loop supply chain with new processes and technologies for personalized therapies. The National Marrow Donor Program (NMDP) has a process for stem cell transplant material, but that is not an FDA-approved product and standards are needed on processes where they do not exist today (e.g., labeling). In order to manage the volume of new therapies, the FDA and the Center for Biologics Evaluation and Research will need many more skilled people to validate and monitor systems.

Therapies require manipulation of cells and extensive quality control tests. Personalized therapies often have long lead times; for example, CAR-T cell therapies take weeks from “vein to vein.” For certain applications, membrane disruption or other methods may hold the promise for decreased time. In addition, next-generation production approaches, potentially from other industries that rigorously manage each process step, should be employed early by manufacturers.

Manufacturing costs per patient in clinical trials are reported to be $100,000 to $300,000 [1, 29, 30] or more for personalized, autologous, ex vivo cell therapies, most of which is driven by overhead and personnel costs. While cost of goods sold (COGS) may be less for other forms of cell and gene therapies, novel and specialized manufacturing processes make scaling to meet commercial demand a significant challenge for all, as evidenced by Novartis’ reported challenges with tisagenlecleucel manufacturing [31]. Efficiently scaling may be a function of the number and size of suites, the time to produce a patient’s medicine, the degree of automation, and the availability of qualified operators. The FDA started a proactive effort to improve manufacturing for gene therapy viral vectors [1]; however, the market will drive solutions in the long term through the early involvement of researchers in developing what will become clinical—and eventually commercial—processes.

### Step 7: Initial and Ongoing Care

Cell therapies are introducing new adverse events with novel mechanisms, and the long-term implications must be studied to improve patient care. A comprehensive review of the safety of cell and gene therapies is outside the scope of this review but should be conducted to help guide physicians. Regardless of treatment type, expanding access will require helping physicians through the initial phases of care, and establishing cross-product registries would benefit long-term patient safety.

Expanding access will require more physicians to gain the capabilities to administer cell therapies; however, the pace of innovation and size and scope of clinical trials means few physicians have actual hands-on experience. Cytokine release syndrome (CRS), which is associated with CAR-T cell therapies, is an example of a potentially lethal adverse event that presents a new challenge. Many hospitals restrict physicians’ ability to engage with manufacturers on commercial products, and regulations restrict commercial interactions to a product’s label. Regulators, manufacturers, hospitals, and academic institutions need to work together to create mechanisms whereby learnings can be disseminated quickly, as patients and physicians may need help in real time.

Gene therapy patients must be followed for 15 years, and the FDA may require follow-up for many other types of cell therapies, a process that will improve the safety of products over time. Traditionally, manufacturers have had separate registries by product, which does not facilitate learning across classes of therapies. To increase knowledge and benefit patient safety long term, existing registries like the Center for International Blood and Marrow Transplant Research (CIBMTR) should be expanded, or new consortiums should be established.

## Challenges for all Cell and Gene Therapies

**Fig. 3:**
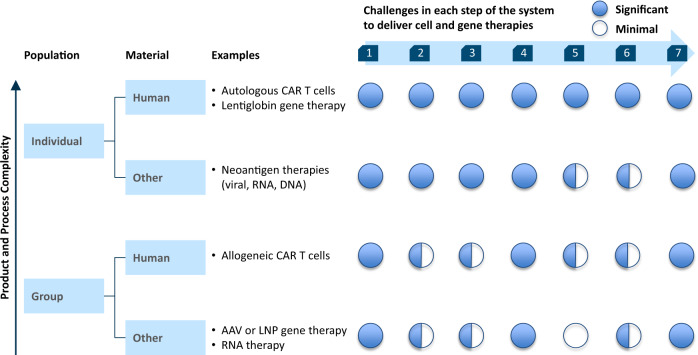
Change needed to deliver cell and gene therapies by system step and therapy type. Many of the systematic challenges to deliver individualized, autologous, ex vivo cell, and gene therapies will also apply to other types of cell and gene therapies. A high-level assessment of the degree of challenges faced at each of the seven steps is provided for types of therapy by population served and material source, though additional work is needed to detail each of these unique challenges

Understanding each step in the system for delivering individual, autologous, ex vivo cell therapies highlights the challenges with forcing these medicines into a system designed for pills or biologics. However, many of these same challenges will also apply to other categories of therapies, as identified in Fig. 3. The degree of challenge for each specific therapy will differ—for example, whether cells are expanded ex vivo or in vivo—but it is clear that systemic changes are needed.

## Stakeholder Changes Required: A Call to Action

We have established a framework for diagnosing and discussing the systemic challenges for delivering cell and gene therapies in a system built for pills and biologics. The framework should be evolved as real-world application reveals more challenges and opportunities; however, the case for change is clear. With many medicines progressing toward approval, and the FDA receiving more than 100 new gene therapy drug applications a year [1], all stakeholders must act immediately.

**Government oversight (FDA, OIG): remove barriers.** The FDA has actively initiated change: for example, in July 2018 it issued a suite of six scientific guidance documents for diseases specific to the manufacturing of gene therapies. Government organizations have a critical responsibility to work with industry groups to establish standards and further remove barriers to manufacturing interactions.

The FDA’s efforts should expand to establishing standards (e.g., REMS, long-term follow-up) by class of product and working with industry groups to add enforcement to self-regulated areas (e.g., FACT accreditation). These efforts will help establish long-term safety and effectiveness, and enable adoption through streamlined processes for provider institutions. Finally, the new role of manufacturers needs to be reflected in protocols and oversight mechanisms for appropriate stakeholder interactions.

**Payers (CMS, state medicaid, private): innovate and clear the path.** CMS must accelerate innovation on new reimbursement mechanisms for cell and gene therapies. A first step to ensuring providers do not have a disincentive to help eligible patients would be to change the inpatient reimbursement approach. CMS must also take the lead providing necessary waivers for innovative mechanisms to align prices with indications and outcomes, including the required operational changes (e.g., systems, codes) to implement such systems at scale.

Private payers have a critical opportunity to accurately value these therapeutics, including covering all health-related costs. Payers must build systems to align price to short- and long-term value by tracking patient outcomes over time, which will likely require cross-payer collaboration.

**Manufacturers: break from your norm.** Manufacturers of cell and gene therapies need a different mindset and process than traditional pharmaceutical and biotechnology companies. Manufacturers should collaborate to ensure consistent standards for incoming material quality and widespread data collection on patient safety within a therapeutic class. Innovative support models to serve as a part of the care team and provide services to patients are needed, and require working with regulators to rewrite the rules rather than adopting old medical-commercial models. Engaging cross-functional teams early in development will ensure fast, cost-efficient, and scalable processes. Finally, manufacturers should innovate pricing structures to align price to value, and work with systems and payers as a part of the solution to implement.

**Industry and nonprofit groups: take the lead and coordinate.** Industry and nonprofit organizations are well-positioned to work across stakeholders to create change. AABB, FACT, CAP, and others should work together to take the lead in creating uniform standards and education by product class. Collaborating with existing organizations (e.g., CIBMTR), manufacturers and providers to develop data-collection mechanisms is another important role.

**Provider organizations: lead the road to scale.** Institutions are on the forefront of delivery and play a critical role in bringing challenges to government, payer, manufacturer, and nonprofit stakeholders to drive executable solutions for standardization and scaling. Institutions should lead the creation of standards for long-term follow-up and monitoring, and develop the model for expert collaboration around safety and efficacy learnings across delivery centers to improve patient care.

**Healthcare professionals and patients: fight for progress.** With all of the challenges and changes needed in our healthcare system, unfortunately action is not likely unless patients, caregivers, patient organizations, and healthcare professionals fight for progress. With many cell and gene therapies targeting rare diseases, coming together to push for these technologies is even more important to ensure that government, regulatory bodies, and payers have these issues on their priority agenda.

We are at the beginning of a revolution in medicine, driven by advances in understanding of humans at a cellular level, along with new therapeutic mechanisms like gene editing. An estimated 30 million people in the U.S. live with one of more than 7000 rare diseases, and 30% of children born with a rare disease will not see their fifth birthday [32]. Thousands and eventually millions of patients stand to benefit from technological advances in potentially transformative ways. Individual solutions are being developed for the few approved therapies, but the pace of innovation requires the rapid and focused development of solutions that can scale the entire delivery system.

This paper reviewed the patient and cell journeys and outlined systemic challenges in order to trigger proactive dialogue between stakeholders to quickly detail and implement solutions for all cell and gene therapies. We must prioritize the changes needed, make “quick wins” happen, and move beyond old systems to ensure that all appropriate patients can benefit from the next generation of medicine.

## Footnotes

1 For example, pembrolizumab, an immuno-oncology medicine, costs $9,870 per dose. The median patient costs $145,233 (10.3 months), and a patient who enters durable remission for 2 years on therapy costs $338,406 (24 months), based on CMS average selling price (ASP) files for October 2018, assuming dose of 200 mg every 3 weeks. Pembrolizumab prescribing information available at: https://www.merck.com/product/usa/pi_circulars/k/keytruda/keytruda_pi.pdf; CMS ASP available at: https://www.cms.gov/Medicare/Medicare-Fee-for-Service-Part-B-Drugs/McrPartBDrugAvgSalesPrice/2018ASPFiles.html
